# Removal and inactivation of human coronavirus surrogates from hard and soft surfaces using disinfectant wipes

**DOI:** 10.1128/aem.01337-25

**Published:** 2025-09-08

**Authors:** Runan Yan, Angela Fraser, Xiuping Jiang

**Affiliations:** 1 Department of Food, Nutrition, and Packaging Sciences, Clemson University 523327, Clemson, South Carolina, USA; Centers for Disease Control and Prevention, Atlanta, Georgia, USA

**Keywords:** human coronavirus OC43, bovine coronavirus, disinfectant wipe, disinfection efficacy, surfaces

## Abstract

Disinfectant wipes are widely used to reduce microbial contamination on surfaces, yet there is limited information on how viruses are physically removed or chemically inactivated during wiping. This study aimed to address this gap by comparing the contributions of physical removal and chemical inactivation to overall disinfection efficacy. Glass and vinyl coupons were contaminated with SARS-CoV-2 surrogates, bovine coronavirus (BCoV), or human coronavirus OC43, at an initial titer of 5–6 log TCID_50_/surface with 5% soil load. After air drying, coupons were wiped using one of the following treatments: (i) pre-wetted blank polypropylene wipe, (ii) hydrogen peroxide (H₂O₂)-based disinfectant wipe, or (iii) quaternary ammonium compound (QAC)-based disinfectant wipe. Wiping was performed manually by hand or mechanically using a Gardco Gardner-scrub. The wiping process followed the U.S. Environmental Protection Agency protocol. After a 1 min exposure, residual disinfectant on both coupons and wipes was neutralized separately. Viruses were recovered by sonication for 30 s and quantified using the TCID_50_ assay with HRT-18G cells. Using blank wipe, more virus was transferred to the wipe from glass (23%–59%) than vinyl (21%–30%), while less virus remained on glass (2%–5%) than vinyl (16%–24%). No significant difference in virus titers was observed between hand wiping and machine wiping, either on the surfaces or in the used wipes. Both disinfectant wipes reduced >3 log_10_ TCID_50_ of virus from surfaces, with virus remaining on used wipes below the limit of detection. These results suggest that disinfectant wipes can significantly and rapidly reduce coronavirus contamination and cross-contamination risk.

**IMPORTANCE:**

Surfaces contaminated with respiratory viruses, such as SARS-CoV-2, pose a potential risk for indirect transmission in public and healthcare settings. This study evaluated the effectiveness of disinfectant wipes in reducing two SARS-CoV-2 surrogates from different surface types within a 1 min contact time. Results showed that both hydrogen peroxide (H₂O₂)-based and quaternary ammonium compound (QAC)-based disinfectant wipes reduced infectious virus levels by more than 3 logs. Physical removal of viruses was more efficient on hard, nonporous surfaces (glass) compared to soft, non-porous surfaces (vinyl). No significant difference was observed between hand wiping and mechanical wiping, indicating that standard wiping procedures can be consistently effective regardless of method. Importantly, our findings highlight that disinfectant wipes function through both physical removal and chemical inactivation mechanisms. These data support evidence-based recommendations for surface disinfection practices to mitigate coronavirus contamination and reduce the risk of fomite-mediated viral transmission.

## INTRODUCTION

Environmental sanitation plays a supporting role in limiting the spread of SARS-CoV-2 by reducing viral contamination on surfaces. Viruses can persist on surfaces, making regular treatment with an EPA-approved antimicrobial (i.e., sanitizer or disinfectant) advantageous (1). Surface disinfection can be achieved through various methods, including but not limited to the use of disinfectant wipes (DW), immersion treatments, and spray-and-wipe techniques, depending on the surface type and disinfection goal (2). DWs are ready-to-use (RTU) meaning they deliver a consistent dose of disinfectant with minimal preparation, making them ideal for targeted treatment of high-touch or smaller areas while reducing the risk of user error. A 2014 study determined the use of RTU disinfectant towelette products resulted in a faster disinfection process, higher compliance with disinfection standards, and overall cost savings as compared to traditional disinfection methods (3). The use of DW as a surface disinfection method to prevent the transmission of pathogens can be effective because of two mechanisms: (i) the inactivation of pathogens upon contact with the disinfectant chemicals and (ii) the physical removal of pathogens from the surface to the wipes. Because pathogens can be removed from a surface and transferred to the wipe, the used wipes that do not contain any disinfectant chemicals represent a source of pathogen transmission from contaminated surfaces to uncontaminated surfaces, potentially leading to the spread of the infectious agent (4). As the physical removal of viruses is not always 100%, the leftover virus on the original contaminated surfaces needs to be inactivated by disinfectant chemical residue on that surface. However, using DW could reduce the transmission of diseases, as pathogens can be theoretically inactivated upon contact with the disinfectant agent absorbed in the wipe.

For a disinfectant to be included on the EPA List N and claim efficacy against SARS-CoV-2, it must demonstrate a 3-log_10_ reduction of a human coronavirus within 10 minutes (5). The efficacy of RTU disinfectant chemicals against viruses must be evaluated using the EPA suspension test (6) or the disk carrier test (7). EPA MB-35 was developed to test the antimicrobial efficacy of DW against a yeast *Candida auris* on hard, non-porous surfaces (8), while EPA Method MB-33-00 is used to evaluate efficacy against bacteria on similar surfaces (9). However, no standard EPA efficacy protocol has been established for viruses on soft surfaces. In a previous study, soft surface disinfection was assessed by applying hydrogen peroxide (H_2_O_2_)-based disinfectants via spraying, however without manual wiping (10). In addition, there is always a debate that the pressure applied by hand wiping can vary not only from person to person but also from time to time. This could lead to the inconsistency of virus removal from the surfaces, thus leaving the remaining virus level uncertain, which could potentially lead to inconsistent results. Therefore, efforts have been made to standardize the wiping method to minimize human-related variations. Generally, the standard protocols often suggest standardizing the wiping patterns, wiping time, and the folding of the disinfectant wipes. To standardize the pressure applied to the surface during wiping, machines have been used. For example, measurements using the ASTM E2967-15 test protocol utilized the Wiperator (FiltaFlex, Almonte, ON) (11). Using this instrument, the inactivation and removal have been evaluated for bacteria and viruses, such as *Staphylococcus aureus* (5, 12), *Clostridium difficile* (5), bacteriophage MS2 (13), and Ebola virus and vesicular stomatitis virus (14). However, this protocol was withdrawn in 2024 without replacement for no specific reasons. The EN 16615 is an European standard that is often referred to as the 4-field test (15). It outlines a controlled procedure where four distinct fields on a test surface are contaminated, wiped, and determined for pathogen reduction. However, this test simulated conditions where a relatively large area is contaminated (5 × 5 cm^2^), which is more suitable to represent a large spill contamination. Besides, the wiping procedure is conducted under a constant pressure of 2.5 kg, which is unlikely to fully replicate human variability, such as applying lighter or inconsistent pressure. The Gardco Gardner-scrub was also used to mimic the wiping procedure under standardized pressure and wiping patterns (16). However, its comparability to hand wiping and its impact on virus removal and disinfection efficacy have not been investigated.

 While studies have explored virus removal and surface disinfection using wipes, there has been limited direct comparison of manual and machine-based wiping methods in practical, everyday settings. The current study has two main objectives: (i) evaluate the effect of virus removal from both non-porous hard and soft surfaces through physical wiping action after a blank wiping treatment (BWT) and (ii) compare the extent of virus reduction, including both removal and inactivation, achieved by hand wiping vs the use of a Gardco Gardner-scrub following a disinfectant wiping treatment (DWT). This study aimed to address this gap by directly comparing these methods and providing valuable insight into their relative efficacy in virus disinfection.

## RESULTS

### Preparation of blank disinfectant wipe

To quantify the physical removal of viruses after the wiping process, blank polypropylene wipes pre-wetted in sterile PBS containing 0.2% Tween 80 were used. The liquid absorption was determined by weight change, and the total weight of pre-wetted wipes was compared with H_2_O_2_-based disinfectant wipes, as they were made from the same material by the same manufacturer. After soaking for 24 h, the blank wipes were completely visually wet and gained an average weight of 4.8 ± 0.2 g per wipe after squeezing out excessive liquid. In comparison, the pre-saturated H_2_O_2_-based disinfectant wipes contained approximately 5 g of disinfectant liquid on average according to the manufacturer’s instructions. The weight of the prewetted blank wipes was approximately 96% of the average weight of H_2_O_2_-based wipes (5 g), indicating blank wipes achieved a comparable level of wetness suitable for experimental purposes.

### Cytotoxicity and neutralization optimization of disinfectant wipes

To prevent potential damage to the cells and to accurately quantify the virus inactivation at the designated contact time, the disinfectant residue on carriers and the used wipes were neutralized before the TCID_50_ assay. The optimized neutralization procedures are shown in Table 1. The cytotoxicity from H_2_O_2_-based DW on HRT-18G cell line was neutralized with 1,300 U L^−1^ catalase, followed by PBS washing using Amicon Ultra-4 centrifugal unit according to our previously published study (17). However, the cytotoxicity of QAC-based disinfectant wipes in HRT-18G cell line became present after 2–3 days of incubation using infection medium and Pierce detergent removal column according to our previous study (18). To further remove the cytotoxic chemicals, the mixture of QAC-based disinfectant and neutralizer was further washed with an Amicon Ultra-4 unit before being added to the cell culture, which delayed the onset of cytotoxicity to days 4–5. To eliminate cytotoxicity during 7 days of incubation, cells were washed with infection medium once after 1 h of incubation. The neutralization procedures resulted in no adverse effects on the HRT-18G cells over 7-day incubation, and the neutralization methods resulted in more than 80% of the recovery of BCoV and HCoV OC43 comparing to the controls.

**TABLE 1:** Neutralization procedures for each disinfectant wipe

Disinfectant wipe	Listed active ingredients	Neutralizer	Additional wash
Product A (H_2_O_2_-based)	H_2_O_2_ (0.5%)	10% FBS and 1,300 U/mL catalase in PBS	Amicon Ultra-4 unit centrifuge (8,000 *g* × 15 min) and PBS wash three times
Product B (QAC-based)	nAlkyl dimethyl benzyl ammonium chloride (0.425%) n-Alkyl dimethyl ethylbenzyl ammonium chloride (0.425%) Ethanol (7.750%) Isopropanol (12.250%)	Infection medium, followed by Pierce detergent column centrifuge (1,000 *g ×* 2 min)	Amicon Ultra-4 unit centrifuge (8,000 *g ×* 15 min) and PBS wash three times. After 1 h incubation for virus attachment, cells at all dilutions were washed twice after 1 h incubation with medium

### Roughness measurements

Profilometry revealed roughness measurements among the microscope glass, gorilla glass, and vinyl coupons (Table 2). Roughness measurements show gorilla glass was the smoothest surface, with the lowest Sq (0.032 µm), Sa (0.026 µm), and Sv (0.103 µm) values (*P* < 0.05), indicating a finely polished and uniform texture. Microscope glass showed slightly higher roughness, with Sq (0.046 µm), Sa (0.037 µm), and Sv (0.203 µm) values. The microscope glass surface also showed slightly higher peak-to-valley height (Sz = 0.301 µm) compared to gorilla glass (Sz = 0.189). However, there was no significant difference in Sdr between two types of glass. Vinyl, in contrast, showed significantly higher roughness values, with highest Sq (18.176 µm), Sa (14.919 µm), and Sv (59.303 µm) values (*P* < 0.05), and a much more irregular surface as reflected by Sz (102.699 µm) and Sdr (62.335%).

**TABLE 2:** Surface roughness characteristics^*a*^

Coupon	Sq (µm)	Sa (µm)	Sv (µm)	Sp (µm)	Sz (µm)	Sdq	Sdr (%)
Microscope glass	0.046 ± 0.001^b^	0.037 ± 0.001^b^	0.203 ± 0.004^b^	0.098 ± 0.011^a^	0.301 ± 0.012^b^	0.008 ± 0^a^	0.004 ± 0^a^
Gorilla glass	0.032 ± 0.001^a^	0.026 ± 0.001^a^	0.103 ± 0.012^a^	0.086 ± 0.012^a^	0.189 ± 0.021^a^	0.009 ± 0.001^a^	0.005 ± 0.001^a^
Vinyl	18.176 ± 4.355^c^	14.919 ± 3.897^c^	59.303 ± 13.39^c^	43.396 ± 17.332^b^	102.699 ± 23.791^c^	1.584 ± 0.001^b^	62.335 ± 5.693^b^

^*a*^ Data labeled with different superscripts (^a^, ^b^, and ^c^) indicate significant differences within each column (*P* < 0.05).

### Virus removal using a blank wipe

The effect of virus removal was assessed using two wiping procedures (Gardco Gardner-scrub and hand wiping) across three carrier surfaces: microscope glass, Gorilla Glass, and vinyl (Tables 3 and 4). For all conditions, the average input virus ranged between 5.7 and 6.2 log₁₀ TCID₅₀ per carrier.

**TABLE 3:** BCoV recovery from carriers and blank wipes with or without hand or machine wiping^*a*^

Carrier	Average input virus control	Virus left on carrier (log_10_ TCID_50_)		Virus transferred to wipe (log_10_ TCID_50_)	
		Gardco Gardner scrub	Hand wiping	Gardco Gardner scrub	Hand wiping
Microscope glass slide	5.7 ± 0.5^a^	4.2 ± 0.2^a^ (3.0% ± 1.4%)^*b*^	4.2 ± 0.4^a^ (3.7% ± 2.9%)	5.5 ± 0.3^a^ (49.3% ± 24.0%)	5.5 ± 0.1^a^ (55.9% ± 15.3%)
Gorilla glass	5.8 ± 0.5^a^	4.4 ± 0.4^a^ (3.9% ± 3.4%)	4.4 ± 0.3^a^ (4.8% ± 2.9%)	5.4 ± 0.5^ab^ (40.4% ± 14.6%)	5.3 ± 0.4^ab^ (42.4% ± 24.1%)
Vinyl	5.8 ± 0.4^a^	5.1 ± 0.2^b^ (23.5% ± 8.1%)	5.0 ± 0.4^b^ (22.0% ± 15.3%)	5.1 ± 0.1^b^ (20.4% ± 4.7%)	5.3 ± 0.1^b^ (30.0% ± 7.2%)

^*a*^ Data labeled with different superscripts (^a^, ^b^, and ^c^) indicate significant differences within each column (*P* < 0.05).

^*b*^ Percentage recoveries are calculated based on the virus titer determined from the corresponding input virus control.

**TABLE 4:** HCoV recovery from carriers and blank wipes with or without hand or machine wiping^*a*^

Carrier	Average input virus control	Virus left on carrier (log_10_ TCID_50_)		Virus transferred to wipe (log_10_ TCID_50_)	
		Gardco Gardner scrub	Hand wiping	Gardco Gardner scrub	Hand wiping
Microscope glass slide	6.2 ± 0.5^a^	4.5 ± 0.4^a^ (2.1% ± 1.2%)^*b*^	4.4 ± 0.4^a^ (1.8% ± 1.2%)	5.7 ± 0.4^a^ (35.1% ± 24.1%)	5.6 ± 0.4^a^ (27.4% ± 19.6 %)
Gorilla glass	6.2 ± 0.5^a^	4.5 ± 0.3^a^ (2.2% ± 1.4%)	4.6 ± 0.3^a^ (2.6% ± 1.6%)	5.7 ± 0.4^a^ (35.6% ± 23.6%)	5.7 ± 0.4^a^ (22.7% ± 19.9%)
Vinyl	5.7 ± 0.4^a^	4.8 ± 0.6^b^ (16.3% ± 15.4%)	4.9 ± 0.5^b^ (23.3% ± 18.7%)	4.9 ± 0.5^b^ (20.5% ± 18.2%)	5.0 ± 0.3^b^ (22.5% ± 11.2%)

^*a*^ Data labeled with different superscripts (^a^, ^b^, and ^c^) indicate significant differences within each column (*P* < 0.05).

^*b*^ Percentage recoveries are calculated based on the virus titer determined from the corresponding input virus control.

No significant differences in residual virus on the carriers after wiping were observed between the two wiping procedures for either virus. However, surface-dependent differences in residual virus levels were observed, and these varied depending on the virus. For BCoV, vinyl surfaces retained significantly more virus than glass surfaces, with approximately 22%–24% of the input virus remaining on vinyl compared to ≤4.8% on glass (*P* < 0.05). Similarly, for HCoV OC43, vinyl surfaces retained significantly higher viruses (15%–19%) than glass surfaces (2%–3%) (*P* < 0.05).

No significant differences in virus transfer to the used wipes were observed between the two wiping procedures for either virus. For both BCoV and HCoV OC43, surface type had a significant effect on virus transfer to the wipe (*P* < 0.05). Transfer of HCoV-OC43 from hard, non-porous glass surfaces (microscope and Gorilla Glass) was significantly higher than from soft, non-porous vinyl surfaces. Similarly, significantly more BCoV was transferred to the wipe from microscope glass than from vinyl. There was no significant difference in virus transfer from Gorilla Glass or microscope glass to the wipe.

### Virus reduction after using disinfectant wipe

**Fig 1:**
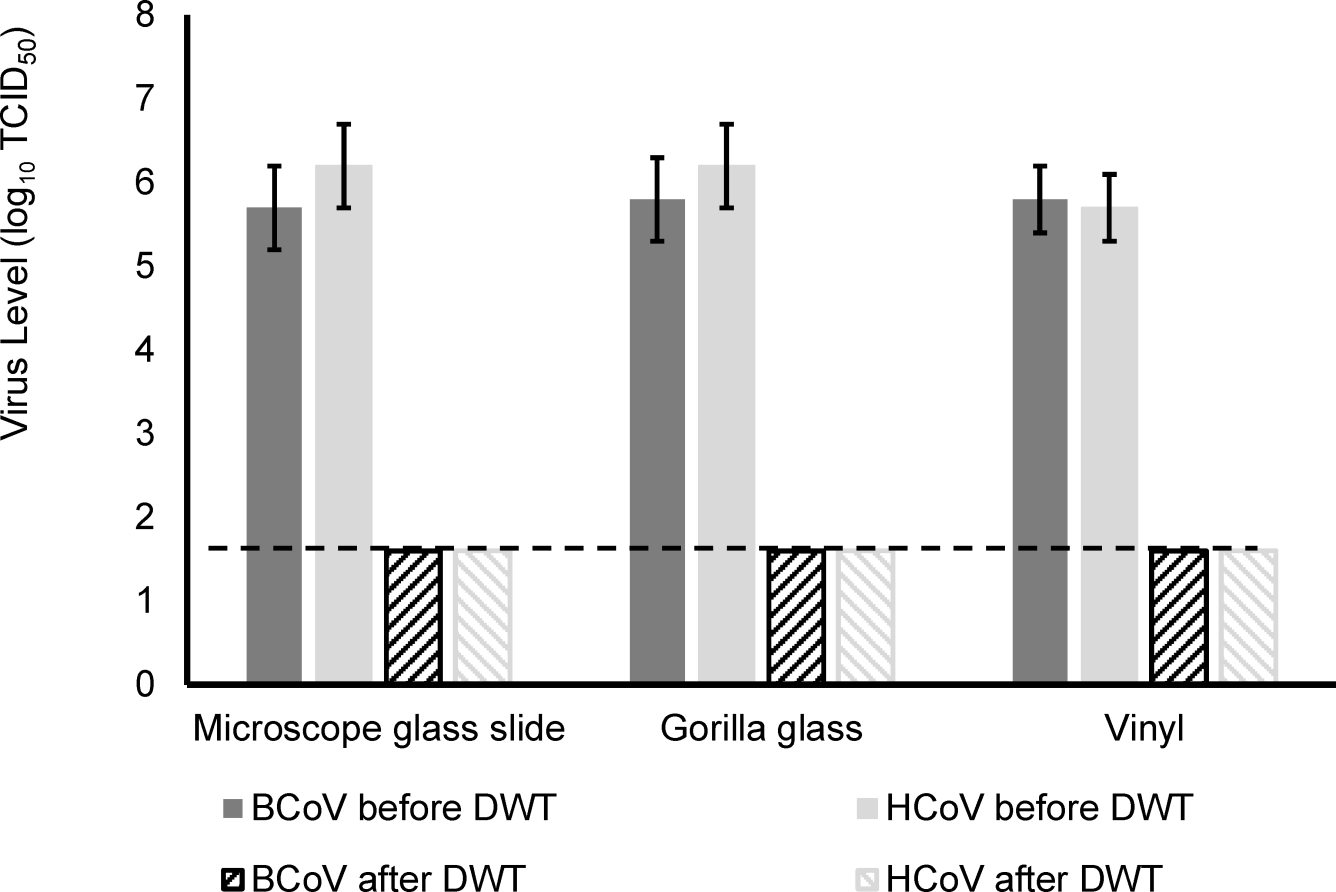
Virus reduction on carriers after a disinfecting wipe treatment (DWT). Solid bars indicate the input virus levels, and the striped bars indicate virus levels that were below the limit of detection on surfaces (dashed line, 1.6 log_10_ TCID_50_ ). Because both wiping methods and all tested surfaces demonstrated a consistent reduction trend, data from all conditions were pooled for presentation. This pooled analysis (*n* = 24) highlights the overall efficacy of two disinfecting wipe treatments across surface types and wiping procedures.

The reduction of BCoV and HCoV was tested using both H_2_O_2_-based and QAC-based wipes, which included (i) virus removal during physical wiping procedures and (ii) the inactivation of viruses by disinfectant. Overall, the reduction of viruses by both wipes was greater than 3 log_10_ TCID_50_ on all carriers (also below the limit of detection, 1.6 log_10_ TCID_50_). The virus reduction was evaluated using both hand wiping and Gardco Gardner scrub, and similar virus reductions (>3 log TCID_50_) were observed for these two wiping procedures. This was consistent across testing with both BCoV and HCoV OC43, where the virus levels were also below the limit of detection following treatment with either wipe type. Since both wiping procedures and all tested surfaces demonstrated a consistent reduction trend, data from all wiping methods and tested surfaces were pooled in Fig. 1 for each virus. This pooled analysis highlights the overall efficacy of the disinfecting wipe treatment across different surfaces and application methods.

## DISCUSSION

This study evaluated the effectiveness of disinfectant wiping as a practical approach for virus removal and inactivation on commonly encountered surface types. We selected two disinfectants with different mechanisms of action: QACs, which disrupt the viral envelope through their detergent properties, and H_2_O_2_, which inactivates viruses by oxidizing proteins, lipids, and nucleic acids. Even though their mechanisms differ, we demonstrated disinfectant wipes containing either H_2_O_2_ or QACs as active ingredients achieved greater than 3-log reductions of coronavirus surrogates on hard, non-porous surfaces (microscope and gorilla glass) and a soft surface (vinyl) using both hand and machine wiping procedures. While effective reductions were also observed on soft, non-porous vinyl, the level of virus removal was lower compared to glass surfaces, suggesting the influence of surface texture on virus removal through wiping. These results highlighted the combined effect of virus physical removal and chemical inactivation in surface disinfection by wiping and provided practical insights into optimizing wiping protocols for different surface materials.

Gorilla glass is a hard, scratch-resistant glass that is used in many electronic products, including smartphones, tablets, and laptops. Therefore, we chose the gorilla glass as it is representative of the surface that many people contact with in their daily life. The microscope glass, on the other hand, is commonly used in the microbiology research labs and was selected for comparison. Our surface characterization data suggest that the gorilla glass was smoother (*P* < 0.05) compared to the microscope glass. However, the virus removal on these two surfaces was not significantly different from each other for both virus surrogates, suggesting that the microscope glass may be used as an alternative testing surface for gorilla glass in similar studies, which is less expensive and easier to obtain when needed. When comparing results to the vinyl carrier, the virus removal from glass surfaces was significantly higher. Because vinyl surfaces are significantly rougher than both glass surfaces, it is reasonable to consider that the roughness of a surface is very important when considering virus removal, among others. Roughness may only play an important role when the difference is large enough to affect the wipe contacting the surfaces, as there was no significant difference in virus removal from the two glass surfaces. It may also be worth considering that surface flexibility (hard vs soft) could influence virus removal, as inconsistent contact between the wipe and the carrier surface may affect the applied physical pressure. Although vinyl was categorized as a “soft” surface in this study due to its flexibility, it is non-porous and, therefore, does not meet the EPA’s definition of a porous “soft surface textile,” which typically includes fabrics and other materials that can absorb liquids. Our intent in including vinyl was to evaluate a flexible, non-porous surface commonly found in institutional and public settings, such as seating or examination table covers.

The EPA published a test to evaluate the virucidal efficacy of pre-saturated or impregnated towelettes for hard, non-porous surface disinfection (single use) against feline calicivirus (FCV), a surrogate virus for Human Norovirus, according to test criteria and methods approved by the US EPA for registration of a product as a virucide (19). In this protocol, it specifies the test wipe should be repeatedly used for 10 times, which may be challenging when the contact time is short, e.g., ≤1 min. The protocol also called for using the blank towelette control for virus removal, such as a gauze or the same material of the disinfectant wipe. In many studies, the blank control testing for virus removal was conducted using a different material, such as J-cloth (14), Tork W8 paper towel (16), commercial detergent wipe (5), and treated polypropylene wipe (1). One concern is virus removal efficacy may be influenced by the wipe material used for wiping. Therefore, in this study, the control blank wipe was made from the same material as the H_2_O_2_-based wipe to allow for an accurate comparison.

As many disinfectant chemicals could potentially damage cell lines, neutralization of these chemicals is needed before carrying out disinfection efficacy tests. Moreover, neutralization of chemicals stops the reaction to allow exact contact time between the disinfectant chemicals and viruses, which will result in accurate and repeatable results. Because different cell lines vary in sensitivity to disinfectant chemicals, and the exact formulation of commercial disinfectants can vary from product to product, it is not possible to find a universal neutralizer for all disinfectant products even though they are labeled with the same active ingredients. It has been reported that H_2_O_2_ can be effectively neutralized by catalase, which accelerates the degradation of H_2_O_2_ into water and oxygen by providing an alternative pathway with a lower activation energy, allowing the decomposition to occur quickly and efficiently (20, 21). In our previous investigation (18), the H_2_O_2_-based RTU disinfectant was neutralized using 1,300 U/mL catalase and 10% FBS suspended in PBS, which helped maintain a neutral pH. However, in the current study, we found that for the H_2_O_2_-based disinfectant wipe, additional filtration using Amicon Ultra-4 centrifugal units was necessary to fully eliminate residual cytotoxic effects. This observation highlights how the composition and absorbency of the wiping material can significantly influence the effectiveness of disinfectant neutralization. Highly absorbent materials may retain larger volumes of disinfectant within their matrix, reducing the disinfectant’s exposure to the neutralizing agent during the recovery step. As a result, neutralization may be incomplete, allowing residual disinfectant activity to persist, particularly for surfactant-based formulations like QACs. Moreover, some wipe materials may release disinfectant gradually or unevenly, leading to localized high concentrations that can remain cytotoxic even after standard neutralization steps. These findings underscore the importance of validating neutralization protocols specifically for wipe-based disinfection assays, considering both the chemical formulation and the physical characteristics of the wiping substrate.

Neutralizing quaternary ammonium compounds (QACs) has remained challenging, as true neutralization is rarely achieved; instead, QAC molecules are typically removed or sequestered (22). Due to their amphiphilic nature, QACs readily form micelles, making the critical micelle concentration (CMC) an important factor when evaluating neutralization buffers (20). Diluting QACs below the CMC may cause micelle disassembly, releasing free QAC molecules and restoring antimicrobial activity. Previous studies have explored various neutralization approaches, including a lecithin and Tween 80 mixture (21, 23, 24). Lecithin was selected for its ability to form micelles that could incorporate QAC molecules and reduce their activity. However, in our study, dilution below the CMC did not fully eliminate cytotoxicity. Moreover, the limited solubility of lecithin resulted in phase separation on the surface of the culture medium, interfering with the microscopic evaluation of cell viability. While lecithin is widely used for neutralizing QACs in bacterial assays, its suitability in viral disinfection assays requires further validation. Filtration-based methods have also been tested for cytotoxicity removal across various detergent types (ionic, anionic, nonionic, and zwitterionic) (25). Among them, the Pierce detergent removal column was shown to eliminate cytotoxicity from three disinfectants while preserving 100% virus recovery. Our previous study also demonstrated the effectiveness of the Pierce column for QAC RTU disinfectant neutralization (18). However, in the current study, residual cytotoxicity against the HRT-18G cell line persisted unless an Amicon Ultra-4 centrifugal filtration unit was incorporated as a final step.

Environmental disinfection is an effective control strategy for healthcare-associated infections (26). Despite numerous products having been manufactured for surface disinfection in healthcare settings, home, or public environments, DW remains a widely used option. Pre-saturated wipes are convenient and easy to use, providing a practical solution across these settings. These pre-saturated disposable wipes, which typically come in small plastic containers with a lid, are designed to be removed one at a time through an opening and discarded after use. On various surfaces, the reduction of pathogens can be achieved using these pre-saturated disinfectant wipes. We used pre-saturated disinfectant wipes to assess virus reduction on surfaces, specifically examining how effectively these wipes remove viral particles and contribute to pathogen control. This approach allowed us to evaluate the efficiency of pre-saturated wipes in achieving surface decontamination, providing valuable insights into their role in preventing the spread of infectious agents in healthcare settings, home, or public environments.

The CDC defines cross-contamination as the transfer of bacteria from one surface to another through direct contact (27). Several studies examined the transfer of pathogens from wipes to uncontaminated surfaces. Lopez et al. reported that *Bacillus thuringiensis* spores, when inoculated on inanimate surfaces, were transferred from disinfected fomites to fingers (28). More recently, Becker et al. demonstrated that disinfectant wipes containing propanol or quaternary ammonium compounds were capable of transferring viruses from a 25 cm² inoculated surface to three other surfaces (29). In our study, the virus load was also quantified from the used disinfectant wipes to determine potential transfer of viruses to the environment. Both BCoV and HCoV OC43 were detected from the blank wipes but not from the disinfectant wipes, suggesting that disinfectant absorbed in wipes may effectively reduce or eliminate virus contamination, preventing transfer to the environment when used properly. Enveloped viruses, such as coronaviruses, are more sensitive to many disinfectants than non-enveloped viruses (30). It has been reported that disinfectant wipes containing 0.75% didecyl-dimethyl-ammonium chloride associated with 0.5% hydrogen peroxide can reduce the alphacoronavirus 1 titer by 3.8 logs on plastic carriers and, thus, can prevent its transmission to secondary surfaces (31). Combined surface disinfection solutions containing 0.5% benzalkonium chloride with laurylamine can reduce the SARS-CoV titer by 6.13 logs after 30 min of exposure, while 0.5% benzalkonium chloride associated with glutaraldehyde and didecyldimonium chloride showed a 3.75 logs reduction in the SARS-CoV titer in 30 min (32).

One of the challenges remained in surfaces disinfection studies using wipes is physical force applied cannot be standardized and, thus, would affect the removal of viruses from the surface. The EPA has given instructions on wiping patterns by standardizing wiping action representative of typical behavior (left-to-right, back-and-forth motion), which gives increased consistency in wiping, while some studies have also used different ways to standardize the force of wiping, such as using the Gardco Gardner-scrub (16), which conducts horizontal reciprocal movement that could mimic the wiping practice, but using a consistent force. In our study, we compared the virus reduction and removal using both hand wiping and the Gardco Gardner scrub. For each virus and each surface type, no significant difference was found in virus removal between hand and machine wiping. The vertical force applied to the surface during hand wiping was approximately 800 g (measured on a scale). For the Gardco Gardner scrub, the weight of the metal wiping head was approximately 450 g. This suggests that differences in applied force beyond 450 g may not result in a measurable difference in virus removal efficiency. Our results clearly indicate that standard wiping procedures can be consistently effective regardless of method. However, the surface areas wiped in clinical or public settings are much larger than those used in this study. Therefore, future research using larger surface areas is warranted to enhance the practical relevance of our findings.

In our study, we used a hydrophobic non-woven wipe pre-saturated with 0.2% Tween 80 to simulate the physical removal of viruses from surfaces using a disinfectant wipe made from the same material. This is because the virus removal can be affected by several factors related to the material, such as its texture, porosity, absorbency, and surface charge (33). Wesgate et al. studied the interaction between two QAC-based disinfectants, a hydrogen peroxide-based product and a neutral cleaner and microfiber, cotton, and nonwoven materials using two standardized test protocols (ASTM E2197-11 and EN 13697-15) for evaluating liquid disinfectants (34). The greatest degree of adsorption (binding) of all products occurred with cotton and microfiber material, and the least with the nonwoven material. Cotton released the smallest amount of disinfectants among the different materials, whereas the nonwoven material released the greatest amount of three of four formulations. However, the overall effect on efficacy of using different wiping materials was limited, except for one of the QAC formulations. Our data also suggest that the virus reduction from a surface after disinfectant wipe treatment would be a combined effect of physical removal and chemical inactivation. However, based on the recovery patterns, physical removal appears to be the predominant mechanism, with chemical inactivation contributing, to a lesser extent, under the tested conditions. These findings are consistent with previous studies using different wiping methods. Following treatment with the acidic anionic surfactants (AAS)-based product, approximately 1 log_10_ lower virus was recovered from the used paper towels compared to those treated with the QAC-based product and the water control, suggesting that the AAS formulation achieved both viral removal and inactivation (16). A comparable observation was made with the NaOCl-based product, where minimal virus was recovered from the used paper towels, even though the same product showed limited virucidal activity in standard ASTM assays conducted without wiping. This suggests that wiping may enhance inactivation efficacy beyond what is observed in static surface tests (16). It should be noted that disinfectant was sprayed on the surfaces, followed by a wiping process in that study. In that case, it is possible that spray application followed by mechanical action during the wiping process itself may assist in resuspension of the viral matrix. While in our study, the contact between virus particle and disinfectant and physical removal occurred almost at the same time. This approach would lead to the immediate removal of viruses, but any residual viral particles remaining on the surface would still require the disinfectant to exhibit its disinfection effect. Therefore, maintaining proper contact time is crucial to ensure the disinfectant can effectively inactivate any remaining viruses on the surface. In the current surface wiping study, this means the wiped surfaces should remain visibly wet before the contact time ends, which may often be neglected during daily use of pre-saturated disinfectant wipes. In practical applications, wiping smaller areas of a surface at a time may help maintain the required wet contact time before drying occurs, particularly for alcohol-containing wipes.

Overall, this research tested the virus removal and reduction during the wiping process using disinfectant wipes, utilizing both hand wiping and a Gardco Gardner-scrub machine wiping procedure. Three types of carriers were used, including two non-porous hard surfaces (microscope glass and gorilla glass) and a soft, non-porous surface (vinyl). Two types of disinfectant chemicals were tested, including an H_2_O_2_-based disinfectant and one QAC-based disinfectant. The blank wiping control was conducted using a heat-melted polypropylene wipe pre-wetted with 0.2% Tween 80. The virus reduction during the wiping procedure was significantly different on vinyl or glass surfaces, suggesting the removal of viruses was less effective on soft surfaces. Using DW with a 1 min contact time, the coronavirus surrogates were reduced by more than 3 logs from both hard, non-porous glass carriers and soft, non-porous vinyl carriers. Moreover, virus surrogates were below the limit of detection on DW after wiping for 1 min, suggesting potential transfer of viruses through the used wipe can be eliminated. Lastly, both hand and machine wiping resulted in a similar trend in virus removal, transmission, and disinfection. This finding highlights the potential for hand wiping, when performed correctly, to be a reliable method for achieving consistent results in virus disinfection studies, comparable to machine-based wiping techniques.

## MATERIALS AND METHODS

### Cell culture

Human rectal tumor (HRT-18G) cells (ATCC CRL-11663) were used to propagate Bovine Coronavirus (BCoV) strain Mebus (BEI Resources NR-445) and Human Coronavirus (HCoV) strain OC43 (ATCC VR-1558). Cells were cultured in T75 vented capped flasks (Corning, Corning, NY) with Dulbecco’s modified Eagle medium, 1× (DMEM; Corning) supplemented with 3% low-endotoxin heat-inactivated fetal bovine serum (FBS; Corning), 100 U L^−1^ penicillin (Corning), and 100 mg L^−1^ streptomycin (Corning) inside a 5% CO_2_ incubator (VWR International, Radnor, PA) at 37°C. HRT-18G cells were subcultured at ~90% confluency (~7 days) in a 1:10 split ratio using 0.25% trypsin EDTA (Thermo Fisher Scientific, Waltham, MA). Cells passaged >30 times were not used for the median tissue culture infectious dose (TCID_50_) assay for virus titration.

### Viral stock preparation

Ninety percent of confluent monolayers of HRT-18G cells were infected with BCoV and HCoV strain OC43 at a multiplicity of infection (MOI) of ~0.05. Initially, the virus had a 1 h adsorption phase with 5 mL infection media (DMEM supplemented with 2% FBS, 100 U L^−1^ penicillin, and 100 mg L^−1^ streptomycin), in a T75 flask containing a monolayer of HRT-18G cells at 33°C and 5% CO_2_. During the viral adsorption phase, the flask was manually rocked every 15 min for even distribution. Then, the infection media was removed from the flask and 15 mL of fresh infection media was added to the flask, followed by incubation for 5–7 days at 33°C until at least ~80% cytopathic effect (CPE). After three freeze-thaw cycles, the cell/virus solution was centrifuged at 5,000 × *g* for 10 min at 4°C (Model 5804 R; Eppendorf, Germany) to remove cell debris. The titers of viral inocula were in the range of approximately 7–8 log_10_ TCID_50_ per mL. The virus stocks were aliquoted and stored at −80°C for the experiments.

### TCID_50_ assay for BCoV and HCoV OC43

HRT-18G cells were seeded with 0.2 mL of cell culture media in 96-well plates (Corning) at a density of ~2.0 × 10^4^ cells per well and incubated at 37°C and 5% CO_2_. HRT-18G plates were used between 90% and 100% confluency (~5 days). Ten-fold serial dilutions of virus/test samples were prepared in infection media in triplicate. Cell culture media was removed from each 96-well plate, and 0.1 mL of the undiluted or serially diluted sample was placed into replicate wells (*n* = 8) of the appropriate 96-well plate and then rocked 10–15 times. The HRT-18G plates were incubated at 33°C and 5% CO_2_ and rocked 10–15 times every 15 min during 1 h. Infection media (0.1 mL) was added to each well, followed by incubation at 33°C and 5% CO_2_ for 7 days, and then scored for CPE. A positive (previous viral stock) and negative control (infection media) were used for each passage of cells. The titers of BCoV and HCoV OC43 were determined via the improved Spearman-Karber method (35). Limit of detection (LOD) for this TCID_50_ assay was calculated as 42 TCID_50_ mL^−1^ (1.6 log_10_ TCID_50_ mL^−1^).

### Cytotoxicity and neutralization test

Cytotoxicity testing and validation of neutralization was conducted using methods outlined in ASTM E1052-20 with modifications (6). To test the cytotoxicity of neutralizers, each neutralizer listed in Table 1 underwent 10-fold serial dilutions in infection media and added to each well containing HRT-18G cells. Experiments were conducted in triplicates. HRT-18G monolayers were observed under an inverted microscope (Leica) for any cytotoxicity after a 1 h, 24 h, and 7 days contact time with undiluted, 10^−1^, and 10^−2^ dilutions. Next, each disinfectant wipe was re-sized aseptically using sterile scissors and then combined with the corresponding neutralizer in a re-sealed whirl-pak bag that accommodated the size of the glass slides (25 × 75 mm^2^). The wipe was then hand massaged for 10 s, followed by sonication for 30 s, and stomached at 230 rpm for 30 s. The wipe was then removed using a sterile tweezer from the whirl-pak bag by squeezing the liquid out, and the liquid mixture underwent 10-fold serial dilutions. The disinfectant/neutralizer mixture was observed for any cytotoxicity against the HRT-18G cell line as described above. If cytotoxicity was observed, an additional “washing step” with infection medium was used to help remove residual antimicrobials that would cause cytotoxicity or interfere with BCoV and HCoV OC43 infectivity.

### Surface carrier and disinfectant wipe preparation

Microscope glass slides (Fisher Scientific) have dimensions of 25 × 75 mm^2^. Vinyl upholstery fabric (Mayer Fabrics, Indianapolis, IN) and gorilla glass slides (Valley Design Crop Operations Inc, Shirley, MA) were cut into the same size. All carriers were washed with LoSUDS Liquid Glassware Detergent (Bar Maid, Pompano Beach, FL), thoroughly rinsed with deionized (DI) water and dried completely. The carriers were then wrapped in aluminum foil and steam-sterilized at 121°C, 15 psi for 20 min. After sterilization, sterile forceps were used to transfer these carriers to a sterile Petri dish for experiments.

The two disinfectant wipes (Product A and B) were selected based on the following criteria: (i) listed in EPA List N and/or List G (for HuNoV), (ii) different labeled active ingredient (H_2_O_2_ or QAC + alcohol), (iii) ≤1 min contact time, and (iv) readily available. Experiments were conducted using two separate lots of each disinfectant wipe.

### Surface roughness measurements

The roughness of microscope glass, gorilla glass, and vinyl coupons was determined using Olympus LEXT OSL5100 optical profiler (Olympus) with the 20× lens. All new coupons were cleaned first, as described above, before the measurement. Using the optical profiler, each surface measurement was conducted at nine different locations for 644.017 × 643.166 µm^2^ per location. The OLS5100 Analysis application was used to analyze the images acquired through the optical profiler. Only the “spike noise removal” was applied before roughness measurements. Roughness measurements (Sq, Sa, Sv, Sp, Sz, Sdq, and Sdr) were determined. Specifically, “Sq” represents the root mean square value of ordinate values within the imaging area, which is equivalent to the standard deviation of heights. “Sa” is the average roughness over the measurement area, which describes the difference in height of each point compared to the arithmetical mean of the surface. “Sv” is the maximum valley depth within the measurement area. “Sp” is the height of the highest peak within the defined area. “Sz” is defined as the sum of the largest peak height value and the largest pit depth value within the defined area (Sv + Sp). “Sdq” is calculated as a root mean square of slopes at all points in the defined area. “Sdr” is expressed as the percentage of the defined area’s additional surface area contributed by the texture as compared to the planar definition area.

### Quantitative carrier test

The antimicrobial efficacy of disinfectant wipes was evaluated against BCoV and HCoV OC43 according to the EPA method on evaluating the virucidal efficacy of pre-saturated or impregnated towelettes for hard, non-porous surface disinfection (single use) against Feline Calicivirus (FCV) with modifications (19). First, 100 µL of BCoV or HCoV OC43 virus suspension (ca. 7.5 log_10_TCID_50_/mL) was inoculated on the center of the carrier. The inoculum was spread with a sterile cell scraper for vinyl carrier due to the hydrophobicity of the surface. While on glass surfaces, inoculum was automatically spread and, thus, cell scrapers were not used. The inoculated carriers were kept inside the biosafety cabinet until visual dryness of the virus inoculum for about 1 h. Once dried, the carriers were wiped using the disinfectant wipe either manually or with the Gardco Gardner-scrub (Gardco), performing three cycles of six passes within 5 s. For manual wiping, the disinfectant wipe was first folded and then used to wipe the carrier under laboratory conditions outside the biosafety cabinet, while wearing sterile gloves. To prevent the accelerated drying of disinfectant due to air-flow and simulate real-world application, the wiped carriers were removed from biosafety cabinet and kept at the ambient laboratory condition for 1 min of contact time, based on the manufacturer’s instructions for use, and then transferred into 10 mL of corresponding neutralizer in a whirl-pak bag wearing sterile gloves, which was resized using a heat sealer to accommodate the size of the carrier so that it can be completely submerged in the neutralizer. The disinfectant wipe was aseptically cut to keep only the area contacted with the carrier during the wiping and then transferred into 10 mL neutralizer in a resized whirl-pak stomach bag. Both the carrier and the wipe were initially hand-massaged for 5 s, followed by sonication for 30 s. The carrier and the wipe were then removed from the bag, and the bag was stomached for 30 s at 230 rpm. One milliliter of the neutralized solution was then washed either using an Amicon Ultra-4 unit (Millipore) or a Pierce detergent removal column (Thermo Scientific) to remove the disinfectant residues (Table 1). After the removal of residual disinfectant, samples were collected and used for titration via TCID_50_ assay with HRT-18G cells, as previously described. To reduce the LOD and increase the dynamic range for efficacy testing, 100 µL of inoculum was used for the TCID_50_ assay. Three replicates of 10-fold serial dilutions of each antimicrobial were tested in two independent experiments. Antimicrobials were considered efficacious if a ≥ 3.0 log_10_ reduction in virus titer was achieved (36).

The input virus control (IVC) served as a positive control, representing the maximum recoverable virus titer from the carrier surface. This allowed us to establish a baseline level of virus titer on the carrier surface and account for any losses inherent to the experimental procedure. Specifically, viruses were inoculated and eluted following the same procedure without any wiping step. To consider the potential loss of over-drying during the experiment, IVC was sampled both before and after the wiping experiment in three replicates, and the average of all six replicates was used as the baseline virus level for the experimental trial. Blank polypropylene wipes (Solenis Inc.) were used as blank wiping control (BWC) to quantify the virus removal due to physical force from the wiping procedures. Due to the hydrophobic nature, the wipes were wetted by soaking in sterile PBS containing 0.2% Tween 80 for 24 h prior to use. Excess liquid was removed from the blank wipe by hand squeezing wearing sterile gloves.

### Statistical analysis

Statistical analysis of virus recovery data from carriers was performed in R using one-way analysis of variance (ANOVA) and Student’s *t* test (*α* = 0.05). Statistical significance was defined as a *P* value of < 0.05.
